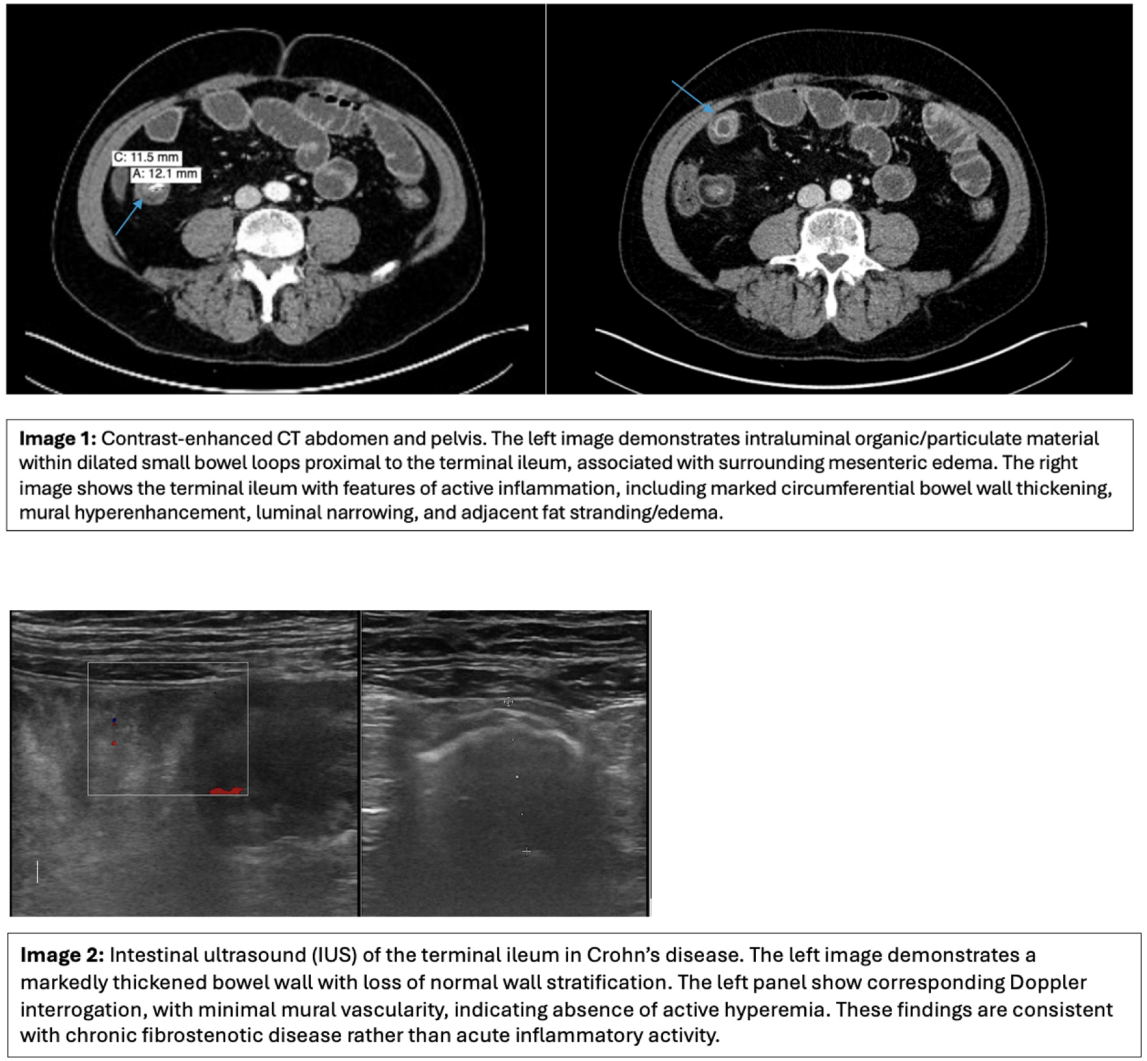# Poster Session I - A150 A RARE CASE OF PARTIAL SMALL BOWEL OBSTRUCTION FROM PHYTOBEZOAR MIMICKING CROHN’S DISEASE FLARE

**DOI:** 10.1093/jcag/gwaf042.150

**Published:** 2026-02-13

**Authors:** R Ahmed, T Guzowski

**Affiliations:** Medicine, University of Saskatchewan, Saskatoon, SK, Canada; Medicine, University of Saskatchewan, Saskatoon, SK, Canada

## Abstract

**Background:**

Phytobezoars are accumulations of indigestible plant material typically found in the stomach or small intestine. While rare, they occur in approximately 4% of all cases of mechanical bowel obstruction. However, this may be an underestimation. Usually described as ovoid or mottled appearing on CT scan. Rarely, they have been described as linear opaque material. Patients with Crohn’s disease develop an environment for phytobezoars to form. There have been observations of a transient increase in inflammatory markers. The diagnostic challenge arises when patients with CD present acutely with symptoms mimicking a flare, but are actually experiencing a mechanical obstruction.

**Aims:**

Intestinal Ultrasound (IUS) in assisting with diagnostic challenges in CD patients with symptoms mimicking a flare, but are actually experiencing a mechanical obstruction.

**Methods:**

A 65-year-old male with quiescent Crohn’s ileitis presented to the Saskatoon Hospital with sudden-onset, non-radiating periumbilical abdominal pain. He had nausea, emesis, and loose, small, infrequent bowel movements. His dietary history revealed a recent increase in high-fiber foods. Previous intestinal ultrasounds had consistently shown minimal scarring of the terminal ileum without signs of active inflammation or critical stenosis.

Upon examination, abdomen was diffusely tender but non-peritonitic. Labs revealed a CRP of 69 mg/L, calprotectin 613 µg/g, and the rest were within normal limits. CT abdomen and pelvis revealed severe mural thickening and submucosal edema of the terminal ileum with an obstructive radiopaque linear foreign body.

The patient was initially managed for presumed CD flare, followed by eventual nasogastric decompression and bowel rest. IUS showed terminal ileum wall thickening with submucosal hypertrophy but preserved wall stratification and normal vascularity. The mesentery was normal, and there were no abnormal loops or signs of penetrating disease.

**Results:**

This patient’s IUS from prior years consistently demonstrated fibrotic segments of the terminal ileum. Bowel wall thickness is often attributed to active inflammation; however, without associated Doppler and mesenteric changes, the bowel wall thickness is likely fibrotic. The rapid normalization of CRP and fecal calprotectin and imaging finding of a radiopaque linear mass in the terminal ileum on CT strongly point toward a transient mechanical obstruction from a phytobezoar, rather than a true inflammatory flare.

**Conclusions:**

These findings align with previous studies where phytobezoars appear as linear organic shadows and can trigger non-specific systemic inflammatory responses in the absence of active IBD. IUS has a vital role in assisting the gastroenterologist in differentiating acute flare from chronic changes in the mucosa in patients presenting with partial obstructions and symptoms of flare.

**Funding Agencies:**

None